# Improved results on nonlinear perturbed T–S fuzzy systems with interval time-varying delays using a geometric sequence division method

**DOI:** 10.1186/s40064-016-2632-4

**Published:** 2016-07-04

**Authors:** Hao Chen

**Affiliations:** College of Electrical and Information Engineering, Southwest University for Nationalities, Chengdu, 610041 China

**Keywords:** Delay-partitioning, Geometric sequence division, Interval time-varying delays, Nonlinear perturbations, T–S fuzzy systems

## Abstract

This paper presents improved stability results by introducing a new delay partitioning method based on the theory of geometric progression to deal with T–S fuzzy systems in the appearance of interval time-varying delays and nonlinear perturbations. A common ratio $\alpha$ is applied to split the delay interval into multiple unequal subintervals. A modified Lyapunov–Krasovskii functional (LKF) is constructed with triple-integral terms and augmented factors including the length of every subintervals. In addition, the recently developed free-matrix-based integral inequality is employed to combine with the extended reciprocal convex combination and free weight matrices techniques for avoiding the overabundance of the enlargement when deducing the derivative of the LKF. Eventually, this developed research work can efficiently obtain the maximum upper bound of the time-varying delay with much less conservatism. Numerical results are conducted to illustrate the remarkable improvements of this proposed method.

## Background

Over the past few decades, complex mathematical modelling with higher order is frequently encountered in many engineering applications, which may cause nonlinearity in dynamic systems. The T–S fuzzy theory introduced in Takagi and Sugeno (1985) can be flexibly applied to approximate the complex nonlinear systems into a unified framework (Zeng et al. 2014; Chang et al. 2015; Chang and Wang 2015; Balasubramaniam et al. 2012; Tanaka et al. 2003). Due to material transfer requirement, accumulation of time lags through system connections and process time, time delays commonly exist in dynamics systems such as chemical processes, communication networks and biological systems, which is considered as a source of instability. Stability analysis of time delayed T–S fuzzy systems has thus been paid special attention (Wu et al. 2011; Zhang et al. 2015b; Zhao et al. 2009).

Stability conditions are classified into two categories delay-independent and delay-dependent. As much of information on the delay is concerned, the delay-dependent criteria is more useful to produce less conservative results (Yang et al. 2015b; Senthilkumar and Mahanta 2010; Lam et al. 2007). Delay partitioning technique, alternatively known as a delay fractionizing method, was developed in Gouaisbaut and Peaucelle (2006). A number of research works have been developed to prove that delay partitioning approach can significantly enhance the stability conditions to obtain less conservatism as soon as the partitions get thinner (Yang et al. 2015a; Zhao et al. 2009; Wang et al. 2015). In Wang et al. (2015), a secondary partitioning method was proposed to further divide primarily separated intervals into a series of smaller segments, which illustrates good stability results. Nonetheless, the research development requires too many adjustable parameters. It thus cost extra computation burden.

In order to further achieve less conservative results, a number of inequalities methods have been proposed, such as Peng–Park’s inequality, reciprocally convex combination, free-matrix-based inequality, etc, which are employed for the purpose of overabundance reduction of the enlargement of the Lyapunov functionals derivative (Sun et al. 2010; Gyurkovics 2015; Park et al. 2011, 2015; Peng and Han 2011; Zeng et al. 2015a). By introducing both augmented state and integral of the state over the period of the delay, these newly developed techniques can preserve extra items when dealing with the enlargement in bounding the derivative of the LKF comparing to the Jensen’s inequality in Seuret and Gouaisbaut (2013). As a result, tighter bounding inequalities are obtained to reduce the conservatism.

In addition, the presence of nonlinearity can cause poor performance and even instability in engineering systems. Robust stability analysis with the effect of the nonlinear perturbation has been investigated with considerable attention (Zhang et al. 2010, 2015a; Ramakrishnan and Ray 2011). Because of process uncertainties and parameter variations, nonlinear perturbations commonly occur in both current and delayed states Ramakrishnan and Ray (2011). The previously developed techniques for such systems are rarely adaptive for the stability analysis with the appearance of nonlinear perturbations. In this paper, T–S fuzzy systems with interval time-varying delays and nonlinear perturbation are considered for stability analysis. Based on the geometric sequence division, some newly developed inequalities, free weight matrices techniques and the Finsler’s Lemma are also employed for obtaining improved stability criteria. Main contributions of this work are:

1. Based on the recently developed geometric sequence division method on delay partitioning, improved stability criteria is presented.
2. Extended reciprocal convex combination(ERCC) is employed for the less enlargement of bounding the derivative of the augmented LKF which is able to reduce the overabundance when deal with the inequalities in the derivative of the LKF.
3. In terms of the system equation, free weight matrices techniques are applied to reduce the conservatism with respect to each fuzzy rule. Numerical examples are conducted to show that the improved stability conditions are obtained by comparing with some existing results.

*Notations*. ${\mathbb {R}}^n$ and ${\mathbb {R}}^{n\times m}$ denote the n-dimensional Euclidean space and the set of all $n\times m$ real matrices, respectively. *I*(0) is the identity (zero) matrix with appropriate dimension; $A^{\mathrm{T}}$ denotes the transpose, and ${\mathrm {He}}(A)=A+A^{\mathrm{T}}$. The symbol $*$ denotes the elements below the main diagonal of a symmetric block matrix. $\Vert \bullet \Vert$ is the Euclidean norm in ${\mathbb {R}}^{n}$. $C\left( [-\tau _b, 0],{\mathbb {R}}^{n}\right)$ is the family of continuous functions $\varphi$ from the interval $[-\tau _b, 0]$ to ${\mathbb {R}}^{n}$ with the norm $\Vert \varphi \Vert _\tau ={\mathrm {sup}}_{-\tau \le \theta \le 0}\Vert \varphi (\theta )\Vert$. The notation $A >(\ge )B$ means that $A-B$ is positive (semi-positive) definite.

## Problem statements and preliminaries

Considering nonlinear perturbed T–S fuzzy systems with interval time-varying delays, for each $l=1,2,\ldots r$ (*r* is the number of the plant rules), the $l{th}$ rule of this fuzzy model with *r* plant rules are described as follows.

Rule *l*: IF $$z_1(t)$$ is $$M_{l 1}$$ and $$\cdots$$$$z_p(t)$$ is $$M_{l p}$$ THEN

1 $$\begin{aligned}&\dot{x}(t)=A_{l}x(t)+B_{l}x\left( t-{\tau (t)}\right) +C_lf\left( x(t),t\right) +D_lg\left( x(t-{\tau (t)}),t\right) ,\quad t\ge 0\\&x(t)=\varphi (t),\quad t\in \left[ -\tau _b,0\right] \\ \end{aligned}$$

where $x(t)\in {\mathbb {R}}^n$ is the state variable, $z_s(t)$ , $M_{ls }\;(s =1,2,\ldots ,p)$ are premise variables and the related fuzzy sets, respectively. $A_{l},B_{l},C_{l},D_{l}$ are the constant matrices with appropriate dimensions. $\tau (t)$ is the time-varying delay. $f\left( x(t),t\right)$ and $g\left( x(t-{\tau (t)}),t\right)$ are unknown nonlinear perturbations with respect to the current state *x*(*t*) and the delayed state $x\left( t-{\tau (t)}\right)$. $\varphi (t)\in C\left( [-\tau _b, 0],{\mathbb {R}}^{n}\right)$ is the initial function.

Then the fuzzy model can be inferred as:

2 $$\begin{aligned} \dot{x}(t)&=\sum _{l=1}^r h_l(t)\left[ A_{l}x(t)+B_{l}x\left( t-{\tau (t)}\right) +C_{l}f\left( x(t),t\right) +D_{l}g\left( x(t-{\tau (t)}),t\right) \right] \\&=A(t)x(t)+B(t)x\left( t-{\tau (t)}\right) +C(t)f\left( x(t),t\right) +D(t)g\left( x(t-{\tau (t)}),t\right) ,\quad t\ge 0 \\ x(t)&=\varphi (t),\quad t\in \left[ -\tau _b,0\right] \\ \end{aligned}$$

where *r* is the number of fuzzy implications, $h_l(t)=\frac{W_l(t)}{\sum _{l=1}^r{W_l(t)}}$, $W_l(t)=\prod _{s=1}^{p}M_{ls}\left( z_s(t)\right)$ with $M_{ls}\left( z_s(t)\right)$ is the grade of membership of $z_s(t)$ in $M_{ls}$. $A(t)=\sum _{l=1}^r h_l(t)A_{l}$, $B(t)=\sum _{l=1}^r h_l(t)B_{l}$ , $C(t)=\sum _{l=1}^r h_l(t)C_{l}$, $D(t)=\sum _{l=1}^r h_l(t)D_{l}$. For $W_l(t)\ge 0$, $h_l(t)\ge 0$ and $\sum _{l=1}^r h_l(t)=1$ thus holds.

The time-varying delay $\tau (t)$ is considered as the following two cases:

### **Case 1**

$\tau (t)$ is a differentiable function satisfying

3 $$0\le \tau _{a}\le \tau (t)\le \tau _{b}, \; \dot{\tau }(t)<\mu , \quad \forall t\ge 0,$$

### **Case 2**

$\tau (t)$ is a continuous function satisfying

4 $$0\le \tau _{a}\le \tau (t)\le \tau _{b}, \quad \forall t\ge 0,$$

where $\tau _{a},\;\tau _{b}, \;\mu$ are constants.

### **Assumption 1**

$$f(0,t)\equiv 0$$ , $$g(0,t)\equiv 0$$ and

5 $$\left\{ \begin{aligned}&f^{\mathrm{T}}f\le \gamma ^2x^{\mathrm{T}}(t)F^{\mathrm{T}}Fx(t)\\&g^{\mathrm{T}}g\le \beta ^2x^{\mathrm{T}}\left( t-{\tau (t)}\right) G^{\mathrm{T}}Gx\left( t-{\tau (t)}\right) \end{aligned} \right.$$

where $\gamma \ge 0,\;\beta \ge 0$ are known scalars, $F\text { and }G$ are known constant matrices, $\forall x\in {\mathbb {R}}^{n}$, and *f* and *g* are the short expressions of $f\left( x(t),t\right)$ and $g\left( x(t-{\tau (t)}),t\right)$, respectively.

A few lemmas are introduced for stability analysis as follows.

### **Lemma 1**

(Han 2003, 2005) *For*$n\times n$*matrix*$Q>0$, *scalar*$\tau >0$, *vector-valued function*$\dot{x}:\left[ -\tau ,0\right] \longrightarrow {\mathbb {R}}^n$*such that the following integrations are well defined, it holds that*

6 $$\begin{aligned} -\tau \int _{t-\tau }^{t}\dot{x}^{\mathrm{T}}(s)Q\dot{x}(s)\,{\mathrm {d}}s\le \left[ \begin{array}{cc} x^{\mathrm{T}}(t) & x^{\mathrm{T}}(t-\tau )\\ \end{array}\right] \left[ \begin{array}{cc} -Q & Q \\ *& -Q \\ \end{array}\right] \left[ \begin{array}{c} x(t) \\ x(t-\tau ) \\ \end{array}\right] \end{aligned}$$

7 $$\begin{aligned} -\frac{\tau ^2}{2}\int _{-\tau }^{0}\int _{t+\theta }^{t}\dot{x}^{\mathrm{T}}(s)Q\dot{x}(s)\,{\mathrm {d}}s{\mathrm {d}}\theta \le \left[ \begin{array}{cc} \tau x^{\mathrm{T}}(t) & \int _{t-\tau }^{t} x^{\mathrm{T}}(s)\,{\mathrm {d}}s\\ \end{array}\right] \left[ \begin{array}{cc} -Q & Q \\ *& -Q \\ \end{array}\right] \left[ \begin{array}{cc} \tau x(t) \\ \int _{t-\tau }^{t} x(s)\,{\mathrm {d}}s \\ \end{array}\right] \end{aligned}$$

### **Lemma 2**

(Zeng et al. 2015, Free-matrix-based integral inequality) *Letxbe a differentiable function :*$[a,b]\rightarrow {\mathbb {R}}^n$, $Z\in {\mathbb {R}}^{n\times n}$*and*$W_1,W_3\in {\mathbb {R}}^{3n\times 3n}$*be symmetric matrices, and*$W_2\in {\mathbb {R}}^{3n\times 3n}$, $N_1,N_2\in {\mathbb {R}}^{3n\times n}$*satisfying this condition*

$$\left[ \begin{array}{ccc} W_1 &\quad W_2 &\quad N_1 \\ *&\quad W_3 &\quad N_2 \\ *&\quad *&\quad Z \\ \end{array}\right] \ge 0$$

*it holds:*

8 $$-\int _{a}^{b}\dot{x}^{\mathrm{T}}(s)Z\dot{x}(s)\,{\mathrm {d}}s\le \varpi ^{\mathrm{T}}\Omega \varpi$$

$${\it{where}} \varpi =\left[ \begin{array}{lll} {x}^{\mathrm{T}}(b) & {x}^{\mathrm{T}}(a)& \frac{1}{b-a}\int _{a}^{b}x^{\mathrm{T}}(s)\,{\mathrm {d}}s\\ \end{array}\right] ^{\mathrm{T}}$$, $$\Omega =(b-a)\left( W_1+\frac{1}{3}W_3\right) +{\mathrm {He}}(N_1\Lambda _1+N_2\Lambda _2),\;\Lambda _1=\bar{e}_1-\bar{e}_2, \;\Lambda _2=2\bar{e}_3-\bar{e}_1-\bar{e}_2,\;\bar{e}_1=\left[ \begin{array}{lll} I & 0 & 0\\ \end{array}\right] ,\;\bar{e}_2=\left[ \begin{array}{lll} 0 & I & 0\\ \end{array}\right] ,\;\bar{e}_3=\left[ \begin{array}{lll} 0 & 0 & I\\ \end{array}\right]$$.

### *Remark 1*

By introducing both augmented state and integral of the state over the period of the delay, the well known Wirtinger-based inequality was developed with less conservatism comparing to the Jensen’s inequality in Seuret and Gouaisbaut (2013) to reduce enlargement in bounding the derivative of the LKF inequalities. However, due to the unadjustable parameters, the tightest upper bound is rarely to be determined in this development. In fact, this Wirtinger-based inequality is the special case of free-matrix-based integral inequality (8) by setting

$$\begin{aligned} N_1&= \frac{1}{b-a}\left[ \begin{array}{lll} -Z & Z & 0 \\ \end{array}\right] ,\;N_2\;=\;\frac{3}{b-a}\left[ \begin{array}{lll} Z & Z & -2Z \\ \end{array}\right] ,\\ W_1&= N_1Z^{-1}N^{\mathrm{T}}_1,\; W_2=N_1Z^{-1}N^{\mathrm{T}}_2,\;W_3=N_2Z^{-1}N^{\mathrm{T}}_2 \end{aligned}$$

Particularly, a set of slack variables inequality in this inequality can be flexibly adjusted, which provide remarkable extra freedom for the purpose of conservatism reduction.

### **Lemma 3**

(Wang et al. 2015, Extendedreciprocalconvexcombination-ERCC) *For any vectors*$f_1,\ldots ,f_N$*with appropriate dimensions, scalars*$k_i(t)\in [0,1]$, $\sum _{i=1}^N k_i(t)=1$, *and matrices*$R_i>0$, *there exist matrix*$S_{ij}(i=1,\ldots , N-1, j=i+1,\ldots ,N)$*satisfies*

$$\left[ \begin{array}{ll} R_i &\quad S_{ij} \\ *&\quad R_j\\ \end{array}\right] \ge 0$$

*then the following inequality holds:*

$$\displaystyle -\sum _{i=1}^N\frac{1}{k_i(t)}f_i^{\mathrm{T}}R_if_i\le -\left[ \begin{array}{ll} f_1 \\ \vdots \\ f_N \\ \end{array}\right] ^{\mathrm{T}} \left[ \begin{array}{lll} R_1 & \cdots & S_{1,N} \\ *&\ddots & \vdots \\ *& *& R_N \\ \end{array}\right] \left[ \begin{array}{l} f_1 \\ \vdots \\ f_N \\ \end{array}\right]$$

### **Lemma 4**

(de Oliveira and Skelton 2001, Finsler’s Lemma) *Let*$\zeta \in {\mathbb {R}}^n,\Phi =\Phi ^{\mathrm{T}}\in {\mathbb {R}}^{n\times n}$, *and*$B\in {\mathbb {R}}^{m\times n}$*with*$rank(B)<n$. *The following statements are equivalent:*

(i) $$\zeta ^{\mathrm{T}}\Phi \zeta <0,\forall B\zeta =0,\quad \zeta \ne 0$$;
(ii) $${B^\perp }^{\mathrm{T}}\Phi B^\perp <0$$;
(iii) $$\exists {\mathcal {L}}\in {\mathbb {R}}^{n\times m}: \Phi +{\mathrm {He}}(\mathcal {L}B)<0$$;

*where*${B^\perp }\in {\mathbb {R}}^{n\times (n-rank(B))}$*is the right orthogonal complement ofB*.

## Main results

**Fig. 1:**
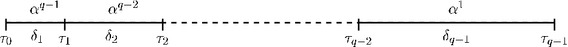
Geometric sequence division based delay partitioning

The stability criteria of T–S fuzzy systems in the presences of interval time-varying delays and nonlinear perturbations are analyzed in this section. In terms of the geometric sequence division method, a new delay partitioning technique is proposed in Fig. 1.

For any integral $m=q-1\ge 1$, the delay interval $[\tau _a, \tau _b]$ is separated into *m* unequal geometric subintervals as,

9 $$\left\{ \begin{aligned}&\delta _i=\alpha ^{q-i}\\&\tau _i=\tau _0+\sum _{k=1}^{i}\alpha ^{q-k},\quad i=1,2,\cdots ,q-1 \end{aligned} \right.$$

where $\tau _a=\tau _0,\;\tau _b=\tau _{q-1}$, and *m* is the number of segments of interval $[\tau _a, \tau _b]$. It is expressed as $[\tau _a, \tau _b]=\left[ \tau _0, \tau _1\right] \bigcup \nolimits _{i=2}^m\left( \tau _{i-1}, \tau _i\right] \triangleq I_1\cup I_2\cup \ldots \cup I_m$. $\alpha$ is a real positive number, and $\delta _i$ is the length of the *ith* subinterval which equals to $\alpha ^{q-i}$. The following expressions are used for notational simplification.

10 $$e_j=\left[ \begin{array}{lll} \underbrace{0,\cdots ,0}_{j-1}, & I & \underbrace{0,\cdots ,0}_{3m-j+6} \\ \end{array}\right] ^{\mathrm{T}} \in {\mathbb {R}}^{n\times (3m+6)n}, \quad j=1,2,\cdots ,3m+6$$

The augmented vector is defined as,

11 $$\xi (t)=\left[ \begin{array}{lllllllll} \dot{x}^{\mathrm{T}}(t), & x^{\mathrm{T}}(t), & x^{\mathrm{T}}(t-\tau _0), & \eta ^{\mathrm{T}}(t) ,& x^{\mathrm{T}}\left( t-\tau (t)\right) ,& \eta ^{\mathrm{T}}_1(t),&\eta ^{\mathrm{T}}_2(t) ,& f^{\mathrm{T}},&g^{\mathrm{T}} \\ \end{array}\right] ^{\mathrm{T}}$$

where

$$\begin{aligned} \eta (t)&=\left[ \begin{array}{lll} x^{\mathrm{T}}\left( t-\tau _1\right) ,& \cdots ,& x^{\mathrm{T}}\left( t-\tau _{q-1}\right) \\ \end{array}\right] ^{\mathrm{T}}\\ \eta _1(t)&=\left[ \begin{array}{lll} \int _{t-\tau _0}^{t}x^{\mathrm{T}}(s)\mathrm {d}s,& \cdots ,& \int _{t-\tau _{q-2}}^{t}x^{\mathrm{T}}(s)\mathrm {d}s\\ \end{array}\right] ^{\mathrm{T}}\\ \eta _2(t)&=\left[ \begin{array}{lll} \frac{1}{\delta _1}\int _{t-\tau _1}^{t-\tau _0}x^{\mathrm{T}}(s)\mathrm {d}s,& \cdots ,&\frac{1}{\delta _{q-1}}\int _{t-\tau _{q-1}}^{t-\tau _{q-2}}x^{\mathrm{T}}(s)\mathrm {d}s \\ \end{array}\right] ^{\mathrm{T}} \end{aligned}$$

Next, the new delay dependent stability criteria is presented for the T–S fuzzy system described in (2).

### **Theorem 1**

*Given a positive integer m, and *$\delta _i=\alpha ^{q-i}$.* Consider (*3)* with time-varying delay satisfying Case 1.  The system* (2)* is asymptotically stable if there exist symmetric positive definite matrices*$Z_i, Q_i,\widetilde{Q}, R_{2i}, R_{3i} \in {\mathbb {R}}^{n\times n} (i=1,2, \ldots , m)$, ${\mathcal {P}}=\left[ P_{ij}\right] _{(m+1)\times (m+1)}\in {\mathbb {R}}^{(m+1)n\times (m+1)n}$, *symmetric matrices *$W_1, W_3 \in {\mathbb {R}}^{3n\times 3n}$,*and*$J\in {\mathbb {R}}^{n\times n}$,*matrices*$W_2 \in {\mathbb {R}}^{3n\times 3n}, N_1,N_2 \in {\mathbb {R}}^{3n\times n}$,*and*${\mathcal {Y}}\in {\mathbb {R}}^{(3m+6)n\times n}$,*such that the following LMIs hold*

12 $$\begin{aligned} W_i= \left[ \begin{array}{lll} W_1 &\quad W_2 &\quad N_1 \\ *&\quad W_3 &\quad N_2 \\ *&\quad *&\quad Z_i \\ \end{array}\right] \ge 0 \end{aligned}$$

13 $$\begin{aligned} \Psi _{k,l}+{\mathrm {He}}(\mathcal {Y}\Gamma _l)<0, \qquad l=1,2,\ldots r \end{aligned}$$

where

$$\begin{aligned} \Gamma _l&=A_le^{\mathrm{T}}_2+B_le^{\mathrm{T}}_{m+4}+C_le^{\mathrm{T}}_{3m+5}+D_le^{\mathrm{T}}_{3m+6}-e^{\mathrm{T}}_1 \\ \Psi _{k,l}&=\Psi _1+\Psi _2+\Psi _{3k}+\Psi _4+\Psi _{51}+\Psi _{52}+\Psi _{l,6}+e_1{\mathcal {Z}}e_1^{\mathrm{T}} \end{aligned}$$

$$\begin{aligned} \Psi _1={\mathrm {He}}\left\{ \left[ \begin{array}{l} e^{\mathrm{T}}_2 \\ e^{\mathrm{T}}_{2m+5}\\ \vdots \\ e^{\mathrm{T}}_{3m+4}\\ \end{array}\right] ^{\mathrm{T}} {\mathcal {P}} \left[ \begin{array}{l} e^{\mathrm{T}}_1 \\ \frac{1}{\delta _1}\left( e^{\mathrm{T}}_3-e^{\mathrm{T}}_4\right) \\ \vdots \\ \frac{1}{\delta _{q-1}}\left( e^{\mathrm{T}}_{m+2}-e^{\mathrm{T}}_{m+3}\right) \\ \end{array}\right] \right\} \end{aligned}$$

$$\begin{aligned} \Psi _2&=e_2\widetilde{Q}e_2^{\mathrm{T}}-(1-\mu )e_{m+4}\widetilde{Q}e_{m+4}^{\mathrm{T}}\\&\quad + \left[ \begin{array}{cc} e^{\mathrm{T}}_3 \\ e^{\mathrm{T}}_4 \\ \vdots \\ e^{\mathrm{T}}_{m+2} \\ e^{\mathrm{T}}_{m+3} \\ \end{array}\right] ^{\mathrm{T}} \left[ \begin{array}{ccccc} Q_1 & 0& \cdots & \cdots & 0 \\ *& Q_2-Q_1 & 0 &\cdots &\vdots \\ *& *&\ddots & 0&\vdots \\ *& *&*& Q_{m}-Q_{m-1} & 0 \\ *& *& *&*& -Q_{m} \\ \end{array}\right] \left[ \begin{array}{cc} e^{\mathrm{T}}_3 \\ e^{\mathrm{T}}_4 \\ \vdots \\ e^{\mathrm{T}}_{m+2} \\ e^{\mathrm{T}}_{m+3} \\ \end{array}\right] \end{aligned}$$

$$\begin{aligned} \begin{aligned} \displaystyle \Psi _{3k}&=\sum _{i=1,i\ne k}^{q-1}\left[ \begin{array}{c} e^{\mathrm{T}}_{i+2} \\ e^{\mathrm{T}}_{i+3} \\ e^{\mathrm{T}}_{2m+4+i}\\ \end{array}\right] ^{\mathrm{T}} \Omega _3 \left[ \begin{array}{c} e^{\mathrm{T}}_{i+2} \\ e^{\mathrm{T}}_{i+3} \\ e^{\mathrm{T}}_{2m+4+i}\\ \end{array}\right] \\&\quad + \left[ \begin{array}{c} e^{\mathrm{T}}_{k+2} \\ e^{\mathrm{T}}_{m+4} \\ e^{\mathrm{T}}_{k+3}\\ \end{array}\right] ^{\mathrm{T}} \left[ \begin{array}{ccc} -Z_k & Z_k-J & J \\ *& -2Z_k+J^{\mathrm{T}}+J & Z_k-J \\ *& *& -Z_k \\ \end{array}\right] \left[ \begin{array}{c} e^{\mathrm{T}}_{k+2} \\ e^{\mathrm{T}}_{m+4} \\ e^{\mathrm{T}}_{k+3}\\ \end{array}\right] \end{aligned} \end{aligned}$$

$$\begin{aligned} \begin{aligned} \displaystyle \Psi _4&=\sum _{i=1}^{q-1}\left[ \begin{array}{l} \tau _{i-1}e^{\mathrm{T}}_2 \\ e^{\mathrm{T}}_{m+4+i}\\ \end{array}\right] ^{\mathrm{T}} \left[ \begin{array}{ll} -R_{2i} &R_{2i}\\ *& -R_{2i} \\ \end{array}\right] \left[ \begin{array}{l} \tau _{i-1}e^{\mathrm{T}}_2 \\ e^{\mathrm{T}}_{m+4+i}\\ \end{array}\right] \\&\quad +\sum _{i=1}^{q-1}\delta ^2_i\left[ \begin{array}{l} e^{\mathrm{T}}_2 \\ e^{\mathrm{T}}_{2m+4+i}\\ \end{array}\right] ^{\mathrm{T}} \left[ \begin{array}{ll} -R_{3i} &R_{3i} \\ *& -R_{3i} \\ \end{array}\right] \left[ \begin{array}{l} e^{\mathrm{T}}_2 \\ e^{\mathrm{T}}_{2m+4+i}\\ \end{array}\right] \end{aligned} \end{aligned}$$

$$\begin{aligned} \begin{aligned} \Psi _{51}&=e_2\lambda _1\gamma ^2F^{\mathrm{T}}Fe_2^{\mathrm{T}}-e_{3m+5}\lambda _1Ie_{3m+5}^{\mathrm{T}}\\ \Psi _{52}&=e_{m+4}\lambda _2\beta ^2G^{\mathrm{T}}Ge_{m+4}^{\mathrm{T}}-e_{3m+6}\lambda _2Ie_{3m+6}^{\mathrm{T}} \end{aligned} \end{aligned}$$

$$\begin{aligned} \begin{aligned} \Psi _{l,6}= \left[ \begin{array}{l} e^{\mathrm{T}}_1 \\ e^{\mathrm{T}}_2\\ e^{\mathrm{T}}_{m+4}\\ e^{\mathrm{T}}_{3m+5} \\ e^{\mathrm{T}}_{3m+6} \\ \end{array}\right] ^{\mathrm{T}} \left[ \begin{array}{lllll} -\widehat{N}_1-\widehat{N}_1^{\mathrm{T}} & \widehat{N}_1A_l-\widehat{N}_2^{\mathrm{T}}& \widehat{N}_1B_l& \widehat{N}_1C_l& \widehat{N}_1D_l \\ *& \widehat{N}_2A_l+A_l^{\mathrm{T}}\widehat{N}_2^{\mathrm{T}} & \widehat{N}_2B_l& \widehat{N}_2C_l& \widehat{N}_2D_l \\ *& *&0 & 0&0 \\ *& *&0& 0 & 0 \\ *& *&0&0& 0 \\ \end{array}\right] \left[ \begin{array}{l} e^{\mathrm{T}}_1 \\ e^{\mathrm{T}}_2\\ e^{\mathrm{T}}_{m+4}\\ e^{\mathrm{T}}_{3m+5} \\ e^{\mathrm{T}}_{3m+6} \\ \end{array}\right] \end{aligned} \end{aligned}$$

*with*

$$\begin{aligned} \begin{aligned} \displaystyle \mathcal {Z}&=\sum _{i=1}^{q-1}\delta _i\alpha ^{q-i}Z_i +\sum _{i=1}^{q-1}\frac{1}{4}\tau ^4_{i-1}R_{2i}+\sum _{i=1}^{q-1}\frac{1}{4}(\tau ^2_i-\tau ^2_{i-1})^2R_{3i}\\ \Omega _3&=\delta ^2_i\left( W_1+\frac{1}{3}W_3\right) +\delta _i\mathrm {He}(N_1\Lambda _1+N_2\Lambda _2) \end{aligned} \end{aligned}$$

### *Proof*

For any $t\ge 0$, there should exist an integer $k\in \left\{ 1,2,\ldots , m\right\}$, such that $\tau (t)\in I_k$. The Lyapunov–Krasovskii functional is as follows:

14 $$V\left( x_t,k\right) \mid _{\tau (t)\in I_k}= V_1(x_t)+V_2(x_t)+V_3(x_t,k)+V_4(x_t)$$

where

$$\begin{aligned}&V_1(x_t)=\epsilon ^{\mathrm{T}}(t)\mathcal {P}\epsilon (t)\\&\displaystyle V_2(x_t)=\int _{t-\tau (t)}^{t}x^{\mathrm{T}}(s)\widetilde{Q}x(s)\mathrm {d}s+\sum _{i=1}^{q-1}\int _{t-\tau _i}^{t-\tau _{i-1}}x^{\mathrm{T}}(t)Q_{i}x(t)\mathrm {d}s \\&\displaystyle V_3(x_t,k)=\sum _{i=1}^{q-1}\delta _i \int _{-\tau _i}^{-\tau _{i-1}}\int _{t+\beta }^{t}\dot{x}^{\mathrm{T}}(s)Z_i\dot{x}(s)\,\mathrm {d}s\,\mathrm {d}\beta \\&\displaystyle V_4(x_t)=\sum _{i=1}^{q-1}\frac{\tau _{i-1}^2}{2}\int _{-\tau _{i-1}}^{0} \int _{\theta }^{0}\int _{t+\lambda }^{t}\dot{x}^{\mathrm{T}}(s)R_{2i}\dot{x}(s)\,\mathrm {d}s\,\mathrm {d}\lambda \,\mathrm {d}\theta \\&\qquad+\sum _{i=1}^{q-1}\frac{\tau _i^2-\tau _{i-1}^2}{2}\int _{-\tau _i}^{-\tau _{i-1}}\int _{\theta }^{0} \int _{t+\lambda }^{t}\dot{x}^{\mathrm{T}}(s)R_{3i}\dot{x}(s)\,\mathrm {d}s\,\mathrm {d}\lambda \,\mathrm {d}\theta \end{aligned}$$

with $$\epsilon (t)= \left[ \begin{array}{ll} x^{\mathrm{T}}(t),& \eta _2^{\mathrm{T}}(t) \\ \end{array}\right] ^{\mathrm{T}}$$

The derivative of the Lyapunov functional $V\left( x_t,k\right) \mid _{\tau (t)\in I_k}$ along the trajectory of the perturbed T–S fuzzy system described in (2) is given as:

15 $$\dot{V}\left( x_t,k\right) \mid _{\tau (t)\in I_k}=\dot{V}_1(x_t)+\dot{V}_2(x_t)+\dot{V}_3(x_t,k)+\dot{V}_4(x_t)$$

where

16 $$\dot{V}_1(x_t)=2\epsilon ^{\mathrm{T}}(t)\mathcal {P}\dot{\epsilon }(t)=\xi ^{\mathrm{T}}(t)\Psi _1\xi (t)$$

The derivative of the second term of the $V_2(x_t)$ is derived as

17 $$\begin{aligned} \frac{d}{dt}&\left( \sum _{i=1}^{q-1}\int _{t-\tau _i}^{t-\tau _{i-1}}x^{\mathrm{T}}(t)Q_{i}x(t)\mathrm {d}s\right) =\sum _{i=1}^{q-1}\left( x^{\mathrm{T}}(t-\tau _{i-1})Q_{i}x(t-\tau _{i-1})-x^{\mathrm{T}}(t-\tau _i)Q_{i}x(t-\tau _i)\right) \\ &=x^{\mathrm{T}}(t-\tau _0)Q_1x(t-\tau _0)+x^{\mathrm{T}}(t-\tau _1)(Q_2-Q_1)x(t-\tau _1)+x^{\mathrm{T}}(t-\tau _2)(Q_3-Q_2)x(t-\tau _2)\ldots \\&+x^{\mathrm{T}}(t-\tau _{q-2})(Q_{m}-Q_{m-1})x(t-\tau _{q-2})-x^{\mathrm{T}}(t-\tau _{q-1})Q_{m}x(t-\tau _{q-1}) \\ &=\left[ \begin{array}{c} x(t-\tau _0) \\ x(t-\tau _1) \\ \vdots \\ x(t-\tau _{q-2}) \\ x(t-\tau _{q-1}) \\ \end{array}\right] ^{\mathrm{T}} \left[ \begin{array}{ccccc} Q_1 & 0& \cdots & \cdots & 0 \\ *& Q_2-Q_1 & 0 &\cdots &\vdots \\ *& *&\ddots & 0&\vdots \\ *& *&*&Q_{m}-Q_{m-1} & 0 \\ *& *& *&*& -Q_{m} \\ \end{array}\right] \left[ \begin{array}{c} x(t-\tau _0) \\ x(t-\tau _1) \\ \vdots \\ x(t-\tau _{q-2}) \\ x(t-\tau _{q-1}) \\ \end{array}\right] \end{aligned}$$

Thus,

18 $$\begin{aligned} \begin{aligned} \dot{V}_2(x_t)\le&x^{\mathrm{T}}(t)\widetilde{Q}x(t)-(1-\mu )x^{\mathrm{T}}(t-\tau (t))\widetilde{Q}x(t-\tau (t))\\&+\left[ \begin{array}{c} x(t-\tau _0) \\ x(t-\tau _1) \\ \vdots \\ x(t-\tau _{q-2}) \\ x(t-\tau _{q-1}) \\ \end{array}\right] ^{\mathrm{T}} \left[ \begin{array}{ccccc} Q_1 & 0& \cdots & \cdots & 0 \\ *& Q_2-Q_1 & 0 &\cdots &\vdots \\ *& *&\ddots & 0&\vdots \\ *& *& *& Q_{m}-Q_{m-1} & 0 \\ *& *& *&*& -Q_{m} \\ \end{array}\right] \left[ \begin{array}{c} x(t-\tau _0) \\ x(t-\tau _1) \\ \vdots \\ x(t-\tau _{q-2}) \\ x(t-\tau _{q-1}) \\ \end{array}\right] \\ =&\xi ^{\mathrm{T}}(t)\Psi _2\xi (t) \end{aligned} \end{aligned}$$

The derivative of $V_3(x_t,k)$ is deduced as

19 $$\begin{aligned} \dot{V}_3(x_t)=\dot{x}^{\mathrm{T}}(t)\left( \sum _{i=1}^{q-1}\delta _i\alpha ^{q-i}Z_i\right) \dot{x}(t)- \sum _{i=1}^{q-1}\delta _i \int _{t-\tau _i}^{t-\tau _{i-1}}\dot{x}^{\mathrm{T}}(s)Z_i\dot{x}(s)\,\mathrm {d}s\ \end{aligned}$$

For the case of $\tau (t)\in I_k (1\le k\le m)$, the second term in (19) is deduced as follows

20 $$\begin{aligned} \begin{aligned} -\sum _{i=1}^{q-1}\delta _i&\int _{t-\tau _i}^{t-\tau _{i-1}}\dot{x}^{\mathrm{T}}(s)Z_i\dot{x}(s)\,\mathrm {d}s \\ &=-\sum _{i=1,i\ne k}^{q-1}\delta _i \int _{t-\tau _i}^{t-\tau _{i-1}}\dot{x}^{\mathrm{T}}(s)Z_i\dot{x}(s)\,\mathrm {d}s -\delta _k\int _{t-\tau _k}^{t-\tau _{k-1}}\dot{x}^{\mathrm{T}}(s)Z_k\dot{x}(s)\,\mathrm {d}s \end{aligned} \end{aligned}$$

Applying Lemma 2 to deal with (20), it is obtained

21 $$\begin{aligned} -\sum _{i=1,i\ne k}^{q-1}\delta _i \int _{t-\tau _i}^{t-\tau _{i-1}}\dot{x}^{\mathrm{T}}(s)Z_i\dot{x}(s)\,\mathrm {d}s \le \sum _{i=1,i\ne k}^{q-1}\varpi ^{\mathrm{T}}_{3i}(t)\Omega _3\varpi _{3i}(t) \end{aligned}$$

where $$\varpi _{3i}(t)=\left[ \begin{array}{lll} {x}^{\mathrm{T}}(t-\tau _{i-1})& {x}^{\mathrm{T}}(t-\tau _i)&\frac{1}{\delta _i}\int _{t-\tau _i}^{t-\tau _{i-1}}x^{\mathrm{T}}(s)\,\mathrm {d}s\\ \end{array}\right] ^{\mathrm{T}}$$.

In the case of $i=k$, applying Jensen’s inequality and the extended ERCC in Lemma 3, it is given as,

22 $$\begin{aligned} \begin{aligned}&-\left( \tau _k-\tau _{k-1}\right) \int _{t-\tau _k}^{t-\tau _{k-1}}\dot{x}^{\mathrm{T}}(s)Z_k\dot{x}(s)\,\mathrm {d}s\\&\quad = -\left( \tau _k-\tau _{k-1}\right) \left( \int _{t-\tau (t)}^{t-\tau _{k-1}}\dot{x}^{\mathrm{T}}(s)Z_k\dot{x}(s)\,\mathrm {d}s +\int _{t-\tau _k}^{t-\tau (t)}\dot{x}^{\mathrm{T}}(s)Z_k\dot{x}(s)\,\mathrm {d}s\right) \\&\quad \le -\frac{\left( \tau _k-\tau _{k-1}\right) }{\left( \tau (t)-\tau _{k-1}\right) }\left( \int _{t-\tau (t)}^{t-\tau _{k-1}}\dot{x}^{\mathrm{T}}(s)\,\mathrm {d}s\right) Z_k \left( \int _{t-\tau (t)}^{t-\tau _{k-1}}\dot{x}(s)\,\mathrm {d}s \right) \\&\quad -\frac{\left( \tau _k-\tau _{k-1}\right) }{\left( \tau _k-\tau (t)\right) }\left( \int _{t-\tau _k}^{t-\tau (t)}\dot{x}^{\mathrm{T}}(s)\,\mathrm {d}s\right) Z_k \left( \int _{t-\tau _k}^{t-\tau (t)}\dot{x}(s)\,\mathrm {d}s\right) \\&\quad =-\frac{\left( \tau _k-\tau _{k-1}\right) }{\left( \tau (t)-\tau _{k-1}\right) } \left[ \begin{array}{l} x(t-\tau _{k-1}) \\ x(t-\tau (t)) \\ \end{array}\right] ^{\mathrm{T}} \left[ \begin{array}{ll} Z_k & -Z_k \\ *&Z_k \\ \end{array}\right] \left[ \begin{array}{l} x(t-\tau _{k-1}) \\ x(t-\tau (t)) \\ \end{array}\right] \\&\quad -\frac{\left( \tau _k-\tau _{k-1}\right) }{\left( \tau _k-\tau (t)\right) } \left[ \begin{array}{l} x(t-\tau (t)) \\ x(t-\tau _k) \\ \end{array}\right] ^{\mathrm{T}} \left[ \begin{array}{ll} Z_k & -Z_k \\ *& Z_k \\ \end{array}\right] \left[ \begin{array}{l} x(t-\tau (t)) \\ x(t-\tau _k) \\ \end{array}\right] \\&\quad \le -\eta ^{\mathrm{T}}_0(t)\left[ \begin{array}{lll} Z_k & -Z_k+J & -J \\ *& 2Z_k-J^{\mathrm{T}}-J & -Z_k+J \\ *& *& Z_k \\ \end{array}\right] \eta _0(t) \end{aligned} \end{aligned}$$

where $$\eta _0(t)=\left[ \begin{array}{lll} x^{\mathrm{T}}(t-\tau _{k-1})&x^{\mathrm{T}}(t-\tau (t)) & x^{\mathrm{T}}(t-\tau _k)\\ \end{array}\right] ^{\mathrm{T}}$$.

Then , it follows from (19–22) that

23 $$\begin{aligned} \dot{V}_3(x_t,k) \le \dot{x}^{\mathrm{T}}(t)\left( \sum _{i=1}^{q-1}\delta _i\alpha ^{q-i}Z_i\right) \dot{x}(t)+ \xi ^{\mathrm{T}}(t)\Psi _{3k}\xi (t) \end{aligned}$$

The derivative of $V_4(x_t)$ is presented as

24 $$\begin{aligned} \begin{aligned} \dot{V}_4(x_t)&=\dot{x}^{\mathrm{T}}(t)\left( \sum _{i=1}^{q-1}\frac{1}{4}\tau ^4_{i-1}R_{2i}+\sum _{i=1}^{q-1}\frac{1}{4}(\tau ^2_i-\tau ^2_{i-1})^2R_{3i}\right) \dot{x}(t)\\&\quad - \sum _{i=1}^{q-1}\frac{\tau _{i-1}^2}{2}\int _{-\tau _{i-1}}^{0} \int _{t+\theta }^{t}\dot{x}^{\mathrm{T}}(s)R_{2i}\dot{x}(s)\,\mathrm {d}s\,\mathrm {d}\theta \\&\quad -\sum _{i=1}^{q-1}\frac{\tau _i^2-\tau _{i-1}^2}{2}\int _{-\tau _i}^{-\tau _{i-1}} \int _{t+\theta }^{t}\dot{x}^{\mathrm{T}}(s)R_{3i}\dot{x}(s)\,\mathrm {d}s\,\mathrm {d}\theta \end{aligned} \end{aligned}$$

By using Lemma 1, the last two terms of (24) are deduced as

25 $$\begin{aligned} \begin{aligned} -\sum _{i=1}^{q-1}&\frac{\tau _{i-1}^2}{2}\int _{-\tau _{i-1}}^{0} \int _{t+\theta }^{t}\dot{x}^{\mathrm{T}}(s)R_{2i}\dot{x}(s)\,\mathrm {d}s\,\mathrm {d}\theta \\&\le \sum _{i=1}^{q-1}\left[ \begin{array}{l} \tau _{i-1}x(t) \\ \int _{t-\tau _{i-1}}^{t}x(s)\,\mathrm {d}s \\ \end{array}\right] ^{\mathrm{T}} \left[ \begin{array}{ll} -R_{2i} & R_{2i} \\ *& -R_{2i} \\ \end{array}\right] \left[ \begin{array}{l} \tau _{i-1}x(t) \\ \int _{t-\tau _{i-1}}^{t}x(s)\,\mathrm {d}s \\ \end{array}\right] \\ -\sum _{i=1}^{q-1}&\frac{\tau _i^2-\tau _{i-1}^2}{2}\int _{-\tau _i}^{-\tau _{i-1}} \int _{t+\theta }^{t}\dot{x}^{\mathrm{T}}(s)R_{3i}\dot{x}(s)\,\mathrm {d}s\,\mathrm {d}\theta \\&\le \sum _{i=1}^{q-1}\left[ \begin{array}{l} (\tau _i-\tau _{i-1})x(t) \\ \int _{t-\tau _i}^{t-\tau _{i-1}}x(s)\,\mathrm {d}s \\ \end{array}\right] ^{\mathrm{T}} \left[ \begin{array}{ll} -R_{3i} & R_{3i} \\ *& -R_{3i} \\ \end{array}\right] \left[ \begin{array}{l} (\tau _i-\tau _{i-1})x(t) \\ \int _{t-\tau _i}^{t-\tau _{i-1}}x(s)\,\mathrm {d}s \\ \end{array}\right] \\&\le \sum _{i=1}^{q-1}(\tau _i-\tau _{i-1})^2 \left[ \begin{array}{l} x(t) \\ \frac{1}{\delta _i}\int _{t-\tau _i}^{t-\tau _{i-1}}x(s)\,\mathrm {d}s \\ \end{array}\right] ^{\mathrm{T}} \left[ \begin{array}{ll} -R_{3i} & R_{3i} \\ *& -R_{3i} \\ \end{array}\right] \left[ \begin{array}{l} x(t) \\ \frac{1}{\delta _i}\int _{t-\tau _i}^{t-\tau _{i-1}}x(s)\,\mathrm {d}s \\ \end{array}\right] \end{aligned} \end{aligned}$$

Thus (24) implies that

26 $$\begin{aligned} \dot{V}_4(x_t)\le \dot{x}^{\mathrm{T}}(t)\left( \sum _{i=1}^{q-1}\frac{1}{4}\tau ^4_{i-1}R_{2i}+\sum _{i=1}^{q-1}\frac{1}{4}(\tau ^2_i-\tau ^2_{i-1})^2R_{3i}\right) \dot{x}(t) +\xi ^{\mathrm{T}}(t)\Psi _4\xi (t) \end{aligned}$$

Referring to (5), for any scalars $\lambda _1\ge 0, \lambda _2\ge 0$ , the nonlinear perturbations can be derived as

27 $$\begin{aligned} \begin{aligned}&0\le \lambda _1\left( \gamma ^2x^{\mathrm{T}}(t)F^{\mathrm{T}}Fx(t)-f^{\mathrm{T}}f\right) =\xi ^{\mathrm{T}}(t)\Psi _{51}\xi (t)\\&0\le \lambda _2\left( \beta ^2x^{\mathrm{T}}(t-{\tau (t)})G^{\mathrm{T}}Gx(t-{\tau (t)})-g^{\mathrm{T}}g\right) =\xi ^{\mathrm{T}}(t)\Psi _{52}\xi (t) \end{aligned} \end{aligned}$$

According to the system in (1), with $\widehat{N}_1$ and $\widehat{N}_2$ are defined as $\widehat{N}_1=\sum _{l=1}^{r}h_l(t)\widehat{N}_{1l}$ and $\widehat{N}_2=\sum _{l=1}^{r}h_l(t)\widehat{N}_{2l}$, and $\widehat{N}_{1l},\widehat{N}_{2l}$ are constant matrices. Then it is given as

28 $$\begin{aligned} \begin{aligned} 0&=2\left[ \dot{x}^{\mathrm{T}}(t)\widehat{N}_1+x^{\mathrm{T}}(t)\widehat{N}_2\right] \left[ A_{l}x(t)+B_{l}x\left( t-{\tau (t)}\right) +C_lf\left( x(t),t\right) +D_lg\left( x(t-{\tau (t)}),t\right) -\dot{x}(t)\right] \\ &=\xi ^{\mathrm{T}}(t)\Psi _{l,6}\xi (t) \end{aligned} \end{aligned}$$

Therefore, the following inequality holds

29 $$\begin{aligned} \dot{V}\left( x_t,k\right) \mid _{\tau (t)\in I_k}\le \sum _{l=1}^r h_l(t)\xi ^{\mathrm{T}}(t)\Psi _{k,l}\xi (t) \end{aligned}$$

Using the augmented vector (11) with the simplification expression (10), the T–S fuzzy system (2) is represented as

30 $$\begin{aligned} 0=\sum _{l=1}^{r}h_l(t)\Gamma _l\xi (t) \end{aligned}$$

where $\Gamma _l$ is defined in Theorem 1.

Hence, the asymptotic stability condition for the T–S fuzzy system (2) with interval time-varying delays and nonlinear perturbations is expressed as

31 $$\begin{aligned} \begin{aligned} \sum _{l=1}^{r}h_l(t)\xi ^{\mathrm{T}}(t)\Psi _{k,l}\xi (t)<0\\ \mathrm {subject\, to}: 0=\sum _{i=1}^{r}h_l(t)\Gamma _l\xi (t) \end{aligned} \end{aligned}$$

Consequently, by means of the Lemma 4, there exists a matrix $\mathcal {Y}$ with appropriate dimensions such that the (31) is equivalent to

32 $$\begin{aligned} \sum _{l=1}^{r}h_l(t)\xi ^{\mathrm{T}}(t)\left[ \Psi _{k,l}+\mathrm {He}(\mathcal {Y}\Gamma _l)\right] \xi (t)<0 \end{aligned}$$

As a result, the derivatives of the newly proposed Lyapunov functionals is deduced as $\dot{V}\left( x_t,k\right) \mid _{\tau (t)\in I_k}<0$. It means $\dot{V}\left( x_t,k\right) \mid _{\tau (t)\in I_k}<\rho \Vert x(t)\Vert ^2$ for sufficiently small $\rho >0$. Hence the T–S fuzzy system in (2) is globally asymptotically stable. This completes the proof.

### *Remark 2*

For the absence of perturbation, that is $C(t)=0, D(t)=0$ , then the T–S fuzzy system (2) is simplified as

33 $$\begin{aligned} \begin{aligned} \dot{x}(t)&=A(t)x(t)+B(t)x\left( t-{\tau (t)}\right),\quad t\ge 0\\ x(t)&=\varphi (t),\quad t\in \left[ -\tau _b,0\right] \end{aligned} \end{aligned}$$

This system has been widely studied Zhao et al. (2009), Wu et al. (2011), Zhang et al. (2015b). The stability criterion for the system is stated below.

### **Theorem 2**

*Given a positive integerm*, *and*$\delta _i=\alpha ^{q-i}$. *Consider* (3) *with time-varying delay satisfying*

**Case 1** The system (33) is asymptotically stable if there exist symmetric positive definite matrices $Z_i, Q_i,\widetilde{Q}, R_{2i}, R_{3i} \in \mathbb {R}^{n\times n} (i=1,2\ldots , m)$, $\mathcal {P}=\left[ P_{ij}\right] _{(m+1)\times (m+1)}\in \mathbb {R}^{(m+1)n\times (m+1)n}$, symmetric matrices $W_1, W_3 \in \mathbb {R}^{3n\times 3n}$, and $J\in \mathbb {R}^{n\times n}$, matrices $W_2 \in \mathbb {R}^{3n\times 3n}, N_1,N_2 \in \mathbb {R}^{3n\times n}$, and $\mathcal {Y}\in \mathbb {R}^{(3m+4)n\times n}$, such that the following LMIs hold

34 $$\begin{aligned} \left[ \begin{array}{lll} W_1 &\quad W_2 &\quad N_1 \\ *&\quad W_3 &\quad N_2 \\ *&\quad *&\quad Z_i \\ \end{array}\right] \ge 0 \end{aligned}$$

35 $$\begin{aligned} \widetilde{\Psi }_{k,l}+\mathrm {He}(\mathcal {Y}\Gamma _l)<0,\quad l=1,2,\ldots , r \end{aligned}$$

where $\Gamma _l=A_le^{\mathrm{T}}_2+B_le^{\mathrm{T}}_{m+4}-e^{\mathrm{T}}_1$, $\widetilde{\Psi }_{k,l}=\Psi _1+\Psi _2+\Psi _{3k}+\Psi _4+\widetilde{\Psi }_{l6}+e_1\mathcal {Z}e_1^{\mathrm{T}}$, and $\Psi _1,\; \Psi _2,\;\Psi _{3k},\;\Psi _4,\;\mathcal {Z}$ have been defined in Theorem 1. $\widetilde{\Psi }_{l6}$ can be deduced by removing the perturbed elements $C_lf\left( x(t),t\right)$ and $D_lg\left( x(t-{\tau (t)}),t\right)$ in (28).

### *Proof*

The same Lyapunov–Krasovskii functional candidate (14) for system (33) is selected for stability analysis. The augment vector (11) is modified as

36 $$\begin{aligned} \widetilde{\xi }(t)=\left[ \begin{array}{ccccccc} \dot{x}^{\mathrm{T}}(t), & x^{\mathrm{T}}(t), & x^{\mathrm{T}}(t-\tau _0), & \eta ^{\mathrm{T}}(t) ,&x^{\mathrm{T}}\left( t-\tau (t)\right) ,&\eta _1^{\mathrm{T}}(t) ,&\eta _2^{\mathrm{T}}(t) \\ \end{array}\right] ^{\mathrm{T}} \end{aligned}$$

where $\eta (t), \eta _1(t)$ and $\eta _2(t)$ are defined in Theorem 1. Then following the similar process of the proof of Theorem 1, the asymptotic stability condition for the T–S system (33) is equivalent to

37 $$\begin{aligned} \sum _{l=1}^{r}h_l\widetilde{\xi }^{\mathrm{T}}(t)\left[ \widetilde{\Psi }_k+\mathrm {He}(\mathcal {Y}\Gamma _l)\right] \widetilde{\xi }(t)<0 \end{aligned}$$

This completes the proof.

### **Corollary 1**

*Given a positive integerm*, *and*$\delta _i=\alpha ^{q-i}$. *Considering*$\tau (t)$*is a continuous function in* (4). *Then the system* (2) *is asymptotically stable if thereexist symmetric positive definite matrices*$Z_i,Q_i, R_{2i}, R_{3i} \in \mathbb {R}^{n\times n} (i=1,2\ldots , m)$, $\mathcal {P}=\left[ P_{ij}\right] _{(m+1)\times (m+1)}\in \mathbb {R}^{(m+1)n\times (m+1)n}$, *symmetric matrices*$W_1, W_3 \in \mathbb {R}^{3n\times 3n}$, *and*$J\in \mathbb {R}^{n\times n}$, *matrices*$W_2\in \mathbb {R}^{3n\times 3n}, N_1,N_2 \in \mathbb {R}^{3n\times n}$, *and*$\mathcal {Y}\in \mathbb {R}^{(3m+6)n\times n}$, *such that the following LMIs hold*

38 $$\begin{aligned} \left[ \begin{array}{lll} W_1 &\quad W_2 &\quad N_1 \\ *&\quad W_3 &\quad N_2 \\ *&\quad *&\quad \widetilde{Z}_3 \\ \end{array}\right] \ge 0 \end{aligned}$$

39 $$\begin{aligned} \widehat{\Psi }_{k,l}+\mathrm {He}(\mathcal {Y}\Gamma _l)<0, \qquad l=1,2,\ldots r \end{aligned}$$

*where*$\widehat{\Psi }_{k,l}$*is deduced from*$\Psi _{k,l}$*by replacing*$\Psi _2$*as*

$$\begin{aligned} \widehat{\Psi }_2= \left[ \begin{array}{c} e^{\mathrm{T}}_3 \\ e^{\mathrm{T}}_4 \\ \vdots \\ e^{\mathrm{T}}_{m+2} \\ e^{\mathrm{T}}_{m+3} \\ \end{array}\right] ^{\mathrm{T}} \left[ \begin{array}{ccccc} Q_1 & 0& \cdots & \cdots & 0 \\ *& Q_2-Q_1 & 0 &\cdots &\vdots \\ *& *&\ddots & 0&\vdots \\ *& *& *& Q_{m}-Q_{m-1} & 0 \\ *& *& *&*& -Q_{m} \\ \end{array}\right] \left[ \begin{array}{c} e^{\mathrm{T}}_3 \\ e^{\mathrm{T}}_4 \\ \vdots \\ e^{\mathrm{T}}_{m+2} \\ e^{\mathrm{T}}_{m+3} \\ \end{array}\right] \end{aligned}$$

$\Gamma _l$*are defined in Theorem* 1.

### *Proof*

For the T–S fuzzy system (2) with interval time-varying delays, modify the Lyapunov functionals (14) by setting $\widetilde{Q}=0$, i.e., $\widehat{V}_2(x_t)=\sum _{i=1}^{q-1}\int _{t-\tau _i}^{t-\tau _{i-1}}x^{\mathrm{T}}(t)Q_{i}x(t)\mathrm {d}s$. Then following the similar process of the proof of Theorem 1, the asymptotic stability condition for the T–S system (2) is equivalent to

40 $$\begin{aligned} \sum _{l=1}^{r}h_l\xi ^{\mathrm{T}}(t)\left[ \widehat{\Psi }_{k,l}+\mathrm {He}(\mathcal {Y}\Gamma _l)\right] \xi (t)<0 \end{aligned}$$

This completes the proof.

### *Remark 3*

Both lower and upper bounds of the time-varying delay $\tau (t)$ are concerned in Cases 1 and 2. Actually, it is pointed out that Case 1 is a special case of Case 2, which means less conservative results can be obtained by using Case 1 instead of Case 2 in the case of a differentiable function of $\tau (t)$. Nonetheless, if $\tau (t)$ is not differentiable, Case 2 is able to overcome this issue Peng and Han (2011).

### *Remark 4*

Considering a unit common ratio, i.e. $\alpha =1$ , which means the length of each subinterval is equivalent. Then previous developed research works using the equivalent partition method Hui et al. (2015), Wang and Shen (2012), Zhao et al. (2009) can be considered as a special case of this proposed approach. Therefore, the developed partitioning method is more generalized.

## Numerical example

In this section, numerical examples are conducted to investigate the stability of the T–S fuzzy systems in (2) and (33).

### *Example 1*

Consider the nominal T–S fuzzy systems (33) with the fuzzy rules described in Peng et al. (2011), Zeng et al. (2015b), Liu et al. (2010) as follows:

41 $$\begin{aligned} \begin{aligned}&\text {Rule} \;1: \text {If}\;z_1(t)\; is \pm \pi /2 ,\; \text {then}\quad \dot{x}(t)=A_1x(t)+B_1x\left( t-\tau (t)\right) \\&\text {Rule} \;2: \text {If}\;z_2(t)\; is \pm 0 ,\; \text {then}\quad \dot{x}(t)=A_2x(t)+B_2x\left( t-\tau (t)\right) \end{aligned} \end{aligned}$$

where the parameters widely discussed are given as,

$$\begin{aligned} A_1=\left[ \begin{array}{cc} -2.1 & 0.1 \\ -0.2 & -0.9 \\ \end{array}\right] , B_1=\left[ \begin{array}{cc} -1.1 & 0.1 \\ -0.2 & -0.9 \\ \end{array}\right] , A_2=\left[ \begin{array}{cc} -1.9 & 0 \\ -0.2 & -1.1 \\ \end{array}\right] , B_2=\left[ \begin{array}{cc} -0.9 & 0 \\ -1.1 & -1.2 \\ \end{array}\right] . \end{aligned}$$

In the Rules 1 and 2, the membership function are $h_1(z(t))=\frac{1}{1+\mathrm {exp}\left( -2z(t)\right) }$, $h_2(z(t))=1-h_1(z(t))$.

Considering the lower bound of the time-varying delay $\tau _a=0$, different values of delay derivative rate $\mu$ are selected to obtain the upper bound of $\tau _b$ for comparisons in Table 1.

**Table 1:** Upper bounds of $\tau _b$ for $\tau _a=0$ and different values of $\mu$

Methods	$\mu =0$	$\mu =0.1$	$\mu =0.5$	Unknown
Liu et al. (2010)	3.30	2.65	1.50	0.79
Zeng et al. (2015b) (m=3)	4.37	3.41	1.95	1.77
Lian et al. (2016)	4.35	3.55	2.32	–
Theorem 2 (m = 3)	4.75	4.06	3.18	1.98

**Fig. 2:**
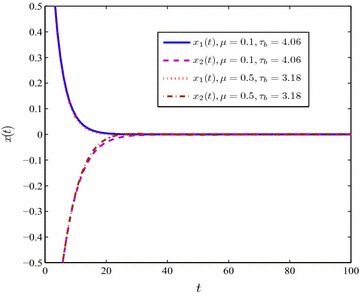
The state response of system (41)

In Table 1, considering different values of $\mu$, the comparisons of the maximum upper bounds $\tau _b$ are given for $\tau _a=0$. According to results in Lian et al. (2016), it is clearly to show that for $\mu =0, \; 0.1, \; 0.5$ this proposed method can dramatically increase the upper bound of the time varying delay when selecting the partitioning number $m=3$. Figure 2 illustrates that with respect to the newly conducted maximum value of $\tau _b$ the state response still converges to zero, which means the T–S fuzzy system (41) is globally asymptotically stable.

Considering $\tau (t)$ to be a continuous function, as it is given in (4), i.e., $\mu$ is unknown. Then upper bound of the $\tau _b$ in this proposed work is compared with some other research results shown in the right column of Table 1.

Referring to the simulation results in Table 1, selecting $\mu =0.1, \, \tau _b=4.06$ and $\mu =0.5, \, \tau _b=3.18$ the state response of the T–S fuzzy system (41) is conducted in Fig. 2.

**Fig. 3:**
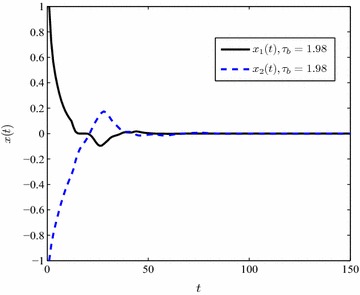
The state response of system (41) with unknown $\mu$

Considering unknown $\mu$, Fig. 3 is shown with $\tau _b=1.98$.

In Figures 2, 3, simulation performance illustrates that under the maximum tolerant delay $\tau _b$ shown in Table 1 the T–S fuzzy system (41) is asymptotical stable.

### *Example 2*

Consider the T–S fuzzy systems (2) in the presence of nonlinear perturbations with the fuzzy rules as follows:

42 $$\begin{aligned} \begin{aligned}&{\text{Rule}\;1}:\; \text {If}\;z_1(t)\; is \pm \pi /2 ,\; \text {then} \quad \dot{x}(t)=A_1x(t)+B_1x\left( t-\tau (t)\right) \\&\qquad +C_1f\left( x(t),t\right) +D_1g\left( x(t-{\tau (t)}),t\right) \\&{\text {Rule}\;2}:\; \text {If}\;z_2(t)\; is \pm 0 ,\; \text {then} \quad \dot{x}(t)=A_2x(t)+B_2x\left( t-\tau (t)\right) \\&\quad +C_2f\left( x(t),t\right) +D_2g\left( x(t-{\tau (t)}),t\right) \end{aligned} \end{aligned}$$

Referring to the Assumption1, system parameters are given as,

$$\begin{aligned} \begin{aligned} A_1=\left[ \begin{array}{cc} -2.1 & 0.1 \\ -0.2 & -0.9 \\ \end{array}\right] , B_1&=\left[ \begin{array}{cc} -1.1 & 0.1 \\ -0.2 & -0.9 \\ \end{array}\right] , A_2=\left[ \begin{array}{cc} -1.9 & 0 \\ -0.2 & -1.1 \\ \end{array}\right] , B_2=\left[ \begin{array}{cc} -0.9 & 0 \\ -1.1 & -1.2 \\ \end{array}\right] , \\&C_1=C_2=D_1=D_2=\left[ \begin{array}{cc} 1 & 0 \\ 0 & 1 \\ \end{array}\right] , \end{aligned} \end{aligned}$$

and $\gamma =\beta =0.1$ In Rules 1 and 2, the membership function are $h_1(z(t))=\frac{1}{1+\mathrm {exp}\left( -2z(t)\right) }$, $h_2(z(t))=1-h_1(z(t))$.

For a given lower bound of $\tau _a=0$ in Theorem 1, considering different values of $\mu$ as well as the unknown $\mu$ in Corollary 1, the upper bounds of $\tau _b$ in this proposed work are obtained in Table 2.

**Table 2:** Upper bounds of $\tau _b$ for $\tau _a=0$ with different values of $\mu$ and unknown $\mu$

Methods	$\mu =0$	$\mu =0.1$	$\mu =0.5$	Unknown
Theorem 1 (m = 3)	3.41	3.29	1.77	1.33
Theorem 1 (m = 4)	4.39	4.28	1.94	1.72

**Fig. 4:**
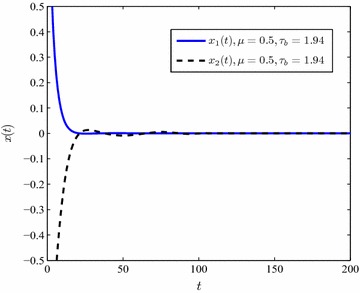
The state response of system (42)

**Fig. 5:**
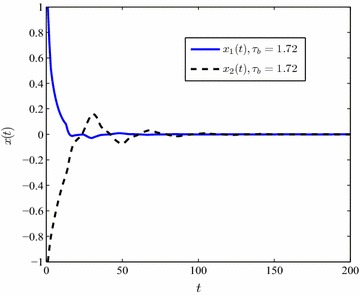
The state response of system (42) with unknown $\mu$

In the presence of nonlinear perturbations, under a fixed value of delay derivative and the unknown $\mu$, the upper bound of delays are conducted in Table 2. It is shown that the proposed method works well in the perturbed T–S fuzzy system (2). By means of the simulation results in Table 2, selecting $\mu =0.5,\,\tau _b=1.94$ and unknown $\mu ,\,\tau _b=1.72$ the state responses of the T–S system (41) are conducted in Figs. 4, 5.

### *Remark 5*

By comparing with the results in Lian et al. (2016), Zeng et al. (2015b), Liu et al. (2010), less conservative results are obtained for the nominal T–S fuzzy system. Simulation results are conducted to demonstrate the remarkable improvements of the proposed method. The proposed geometric progression technique for delay partition can deal with the time-varying delayed T–S fuzzy systems with nonlinear perturbations with excellent stability criteria.

### *Remark 6*

Tables 1 and 2 demonstrate that the maximum value of $\tau _b$ drops down when $\mu$ increases. In addition, the upper bound of time-varying delay $\tau (t)$ becomes bigger as soon as the partitioning segment gets finer. Figures 2, 3, 4 and 5 display that the convergence time of the state response rises up in the case of an unknown delay derivative $\mu$.

## Conclusions 

In this paper, a novel delay partitioning method using the geometric sequence division is proposed for stability analysis of the perturbed T–S fuzzy system with interval time-varying delays. Recently developed inequalities and new modified Lyapunov functionals are introduced in this work. Numerical examples are given to demonstrate that less conservative results can be obtained in this design by comparing with some previously developed approaches.
